# Immunohistochemical Expression of Invadopodia‐Associated Proteins in Subtypes of Unicystic Ameloblastoma

**DOI:** 10.1111/odi.70338

**Published:** 2026-04-06

**Authors:** Gabriela Cristina Avertano Rocha da Silveira, Rebeca Vieira Costa, Flavia Letícia Magalhães Lemos, Antonia Taiane Lopes de Moraes, Maria Sueli da Silva Kataoka, Vanessa Morais Freitas, Douglas Magno Guimarães, Ana Paula Neutzling Gomes, Adriana Etges, Fabricio Passador Santos, Vera Cavalcanti de Araújo, Andrew Mark Smith, Sérgio de Melo Alves Júnior, Ruy Gastaldoni Jaeger, João de Jesus Viana Pinheiro

**Affiliations:** ^1^ Laboratory of Pathological Anatomy and Immunohistochemistry, School of Dentistry Federal University of Pará Belém Pará Brazil; ^2^ Oral Diagnosis Department, Piracicaba Dental School University of Campinas (UNICAMP) Piracicaba São Paulo Brazil; ^3^ Cell Culture Laboratory, School of Dentistry Federal University of Pará Belém Pará Brazil; ^4^ Institute of Biomedical Sciences University of São Paulo São Paulo São Paulo Brazil; ^5^ School of Dentistry University Center of Pará Belém Pará Brazil; ^6^ Center for the Diagnosis of Diseases of the Mouth, School of Dentistry Federal University of Pelotas Pelotas Rio Grande do Sul Brazil; ^7^ Department of Oral Pathology São Leopoldo Mandic Institute and Research Center Campinas São Paulo Brazil; ^8^ Department of Microbial Diseases, Eastman Dental Institute University College London London UK

**Keywords:** ameloblastoma, extracellular matrix, immunohistochemistry, invasion, odontogenic tumors

## Abstract

**Objective:**

This study aims to investigate the expression of invadopodia‐associated proteins—HIF‐1α, NOTCH‐1, ADAM‐12, and HB‐EGF—across different histological subtypes of unicystic ameloblastomas (UA) to assess their invasiveness under hypoxic conditions.

**Materials and Methods:**

Immunohistochemical analysis was performed on 29 UA samples, 9 conventional ameloblastomas (CAM), and 9 dental follicles (DF).

**Results:**

Mural unicystic ameloblastomas (MUA) exhibited significantly higher immunoexpression of all proteins compared to luminal (LUA) and intraluminal (IUA) subtypes (*p* < 0.0001). Neoplastic cells in the cystic capsule of MUA also showed greater expression of these proteins than those in the lumen. Conventional ameloblastomas demonstrated lower expression of NOTCH‐1, ADAM‐12, and HIF‐1α compared to the MUA cystic capsule cells. Dental follicles had the lowest immunostaining of all groups.

**Conclusion:**

These findings suggest that hypoxia may contribute to more aggressive behavior in MUA, potentially enhancing its invasive capacity compared to LUA and IUA.

## 1 Introduction

Unicystic ameloblastoma can account for 5%–22% of ameloblastoma cases and is histologically classified into three types: luminal unicystic ameloblastoma (LUA), intraluminal unicystic ameloblastoma (IUA), and mural unicystic ameloblastoma (MUA) (El‐Naggar et al. 2017; WHO Classification of Tumors Editorial Board 2024). Approximately 50% of cases occur in the second decade of life, with a male predilection (WHO Classification of Tumors Editorial Board 2024). Notably, up to two‐thirds of unicystic ameloblastomas may have a mural component, exhibiting a more aggressive behavior similar to conventional ameloblastomas (El‐Naggar et al. 2017; WHO Classification of Tumors Editorial Board 2024). The World Health Organization's 2024 classification suggests mural unicystic ameloblastoma as an indeterminate category between the conventional and unicystic forms (WHO Classification of Tumors Editorial Board 2024).

Tumor invasion is influenced by hypoxia, which activates compensatory cellular mechanisms, including the formation of invadopodia—actin‐rich protrusions that degrade the extracellular matrix (ECM) (Buccione et al. 2009; Nascimento et al. 2010; Da Costa et al. 2016; Greer et al. 2012). Several key proteins regulate this process, including hypoxia‐inducible factor‐1 alpha (HIF‐1α), Notch homolog 1 (NOTCH‐1), disintegrin and metalloproteinase 12 (ADAM‐12), and heparin‐binding epidermal growth factor (HB‐EGF) (Da Costa et al. 2016). HIF‐1α plays a central role in tumor adaptation to hypoxia by promoting angiogenesis, metabolism, and invasion (Greer et al. 2012; Kilic et al. 2007; Semenza 2003; Courtneidge et al. 2005). ADAM‐12 contributes to tumor growth by cleaving biologically active ligands such as tumor necrosis factor‐alpha (TNF‐α), epidermal growth factor (EGF), and transforming growth factor‐alpha (TGF‐α) and is linked to malignancies like breast and lung cancer (Kveiborg et al. 2005; Rocks et al. 2006; Walsh et al. 2014). HB‐EGF enhances tumor aggressiveness by promoting DNA replication and resistance to growth inhibition (Adam 2010). NOTCH‐1 activation, a key driver of tumor invasion, induces the transcription of multiple genes, including ADAM‐12, and interacts with signaling pathways that regulate cellular differentiation (Díaz et al. 2013; Hagg and Wennstrom 2005; Zhu et al. 2013).

A study on odontogenic tumors found increased expression of NOTCH‐1, HIF‐1α, ADAM‐12, and HB‐EGF in conventional ameloblastomas, particularly in the tumor core and adjacent cystic epithelium. These proteins were linked to intratumoral hypoxia and implicated in invadopodia formation, enhancing tumor invasiveness. The findings suggest that NOTCH‐1 stabilizes HIF‐1α, which then promotes ADAM‐12 and HB‐EGF expression, contributing to local tumor aggression (Da Costa et al. 2016).

Intratumoral hypoxia plays a critical role in regulating tumor invasiveness, offering deeper insights into the molecular mechanisms that could improve diagnostic approaches and guide the treatment of unicystic ameloblastomas. Therefore, this study aims to investigate the expression of invadopodia‐associated proteins—HIF‐1α, NOTCH‐1, ADAM‐12, and HB‐EGF—across different histological subtypes of unicystic ameloblastomas and correlate their expression with the distinct biological behavior of these tumors.

## 2 Material and Methods

### 2.1 Ethical Aspects

This study was conducted in accordance with the criteria established by the Research Ethics Committee on Human Subjects of the Institute of Health Sciences at the Federal University of Pará (ICS/UFPA) and was approved under protocol number 4.570.860, following the Declaration of Helsinki (World Medical Association 2013).

### 2.2 Study Sample

Immunohistochemical reactions were performed on 47 human derived samples: 29 unicystic ameloblastoma (UA) samples, 9 conventional ameloblastoma (CAM) samples, and 9 dental follicles (DF) samples. Patient samples and clinical data were collected from the archives of the Center for Diagnosis of Oral Diseases (CDDB) at the School of Dentistry of the Federal University of Pelotas, the São Leopoldo Mandic Research Center and Institute, and the Laboratory of Pathological Anatomy and Immunohistochemistry at the School of Dentistry of the Federal University of Pará. All samples were diagnosed based on imaging analysis and intraoperative examinations, combined with a histological analysis of the entire lesion, to rule out tumor epithelium invasion into the cystic capsule in UA cases.

The total sample was divided into five groups according to the 2024 WHO classification for head and neck tumors: the MUA group, with 14 samples of the mural subtype; the LUA group, with 7 samples of the luminal subtype; and the IUA group, with 8 samples of the intraluminal subtype. The CAM group consisted of nine samples microscopically diagnosed as conventional ameloblastoma, serving as a positive control due to the established expression of HIF‐1α, NOTCH‐1, ADAM‐12, and HB‐EGF in this tumor. Additionally, the DF group included nine samples of dental follicles, which are normal dental tissues that do not exhibit cystic or neoplastic alterations (WHO).

Following the same diagnostic criteria, all UA and CAM cases were diagnosed based on clinical, radiographic, surgical, and histopathological features.

### 2.3 Immunohistochemistry

For immunohistochemical analysis, the histological slides were deparaffinized in xylene and rehydrated in decreasing concentrations of ethanol. The samples were then immersed in 3% hydrogen peroxide and methanol (1:1) to block endogenous peroxidase activity. Antigen retrieval was performed using citrate buffer (pH 6.0) in a Pascal pressure chamber (Dako Cytomation, Carpinteria, CA, USA) for 30 s at 125°C.

After treatment with 1% bovine serum albumin (Sigma‐Aldrich, St. Louis, MO, USA) in phosphate buffered saline for 40 min, the sections were incubated with the primary antibodies Anti‐NOTCH‐1 (1:4000; Millipore, Burlington, MA, USA) and Anti‐ADAM‐12 (1:100; BiOss, Woburn, MA, USA) for 1 h. Meanwhile, the primary antibodies anti‐HIF‐1α (1:50; Millipore, Burlington, MA, USA) and Anti‐HB‐EGF (1:25; Invitrogen—Thermo Fisher Scientific, Carlsbad, CA, USA) were incubated overnight in a humid chamber at room temperature.

The slides were incubated and treated at room temperature with a dextran polymer based Reveal complex (Spring Bioscience, Pleasanton, CA, USA), and diaminobenzidine (DAB) was used as the chromogenic agent (Liquid DAB + Substrate; Spring Bioscience, Pleasanton, CA, USA). Finally, the slides were counterstained with Mayer's hematoxylin (Sigma‐Aldrich) and mounted with Permount mounting medium (Fisher Scientific, Fair Lawn, NJ, USA).

### 2.4 Immunostaining Evaluation

Five bright field images were randomly acquired from each sample using an AxioScope microscope (Carl Zeiss, Oberkochen, Germany) equipped with an AxiocCam HRC color CCD camera (Carl Zeiss). The images were obtained at the same magnification (400×) and saved in TIFF format. In the cases of MUA, 10 images were acquired per sample: 5 images of cells invading the cystic capsule and 5 images of cells present in the lumen.

The areas stained by diaminobenzidine were analyzed using the “Immunohistochemistry (IHC) Image Analysis Toolbox” (Jie Shu, Guoping Qiu, and Mohammad Ilyas, https://imagej.nih.gov/ij/plugins/ihc‐toolbox/index.html) within the ImageJ software (public domain software version 14), developed by Wayne Rasband (NIMH, NIH, Bethesda, MD, USA, http://rsbweb.nih.gov/ij/).

Immunostaining evaluation was performed by measuring the immunostaining fraction (%) of HIF‐1α, NOTCH‐1, ADAM‐12, and HB‐EGF antibodies in neoplastic cells stained with DAB relative to the total quantified parenchyma. The mean percentage of staining across the five fields per sample was analyzed using GraphPad Prism 5 software (GraphPad Software Inc., San Diego, CA, USA).

### 2.5 Statistical Analysis

The Shapiro–Wilk test was used to assess the normality of data distribution. For parametric data, analysis of variance (ANOVA) was performed, followed by Bonferroni multiple comparison correction. For nonparametric data, the Kruskal–Wallis test, followed by Dunn's multiple comparison correction. The difference between the capsule and lumen areas in MUA was evaluated using the Student's *t*‐test for parametric data and the Mann–Whitney test for nonparametric data. A 95% confidence interval was assumed (*p* = 0.05).

## 3 Results

Samples from different types of ameloblastoma were grouped based on clinical and histopathological data, as shown in Table 1.

**TABLE 1:** Clinical and demographic characteristics of ameloblastoma samples: Histological subtype, sex, age, and anatomical location of the lesion.

	Unicystic ameloblastoma			Conventional ameloblastoma	Dental follicle
	Mural	Luminal	Intraluminal		
Sex (*n* = 47)					
Female	8	5	3	4	4
Male	3	2	4	5	5
NR	3	0	1	0	0
Age (*n* = 47)					
00–09	1	0	2	0	0
10–19	1	3	4	0	5
20–29	7	3	1	1	2
30–39	0	0	0	4	1
40–49	0	0	0	2	0
50–59	0	1	0	2	0
60–69	0	0	0	0	0
70–79	0	0	0	0	0
80–89	0	0	0	0	0
NR	4	0	1	0	1
Anatomical location (*n* = 47)					
Maxilla	1	0	0	0	0
Mandible	9	5	5	10	8
NR	5	1	1	0	2

Abbreviation: NR, not related.

All samples from the CAM, MUA, LUA, IUA, and DF groups exhibited expression of NOTCH‐1, ADAM‐12, HIF‐1α, and HB‐EGF in cells of the neoplastic parenchyma as well as in the epithelium of the DF. The immunohistochemical staining of the studied proteins was localized in the cords and islands of the epithelium of odontogenic tumors and the dental follicle.

MUA exhibited a higher rate of NOTCH‐1 immunoexpression compared to LUA (*p* < 0.0001), IUA (*p* < 0.0001), and CAM (*p* < 0.0001). Neoplastic cells in the cystic capsule of MUA showed a higher percentage of NOTCH‐1 expression compared to the lumen (*p* < 0.0001). NOTCH‐1 staining was predominantly observed in the cytoplasm of the tumor parenchyma cells, although weak nuclear staining was also noted. Histological images and the comparison results of NOTCH‐1 protein between the UA, CAM, and DF groups are shown in Figure 1, while the comparison of the immunostaining rate of this protein between the neoplastic cell regions in the capsule and lumen of MUA can be seen in Figure 2.

**FIGURE 1:**
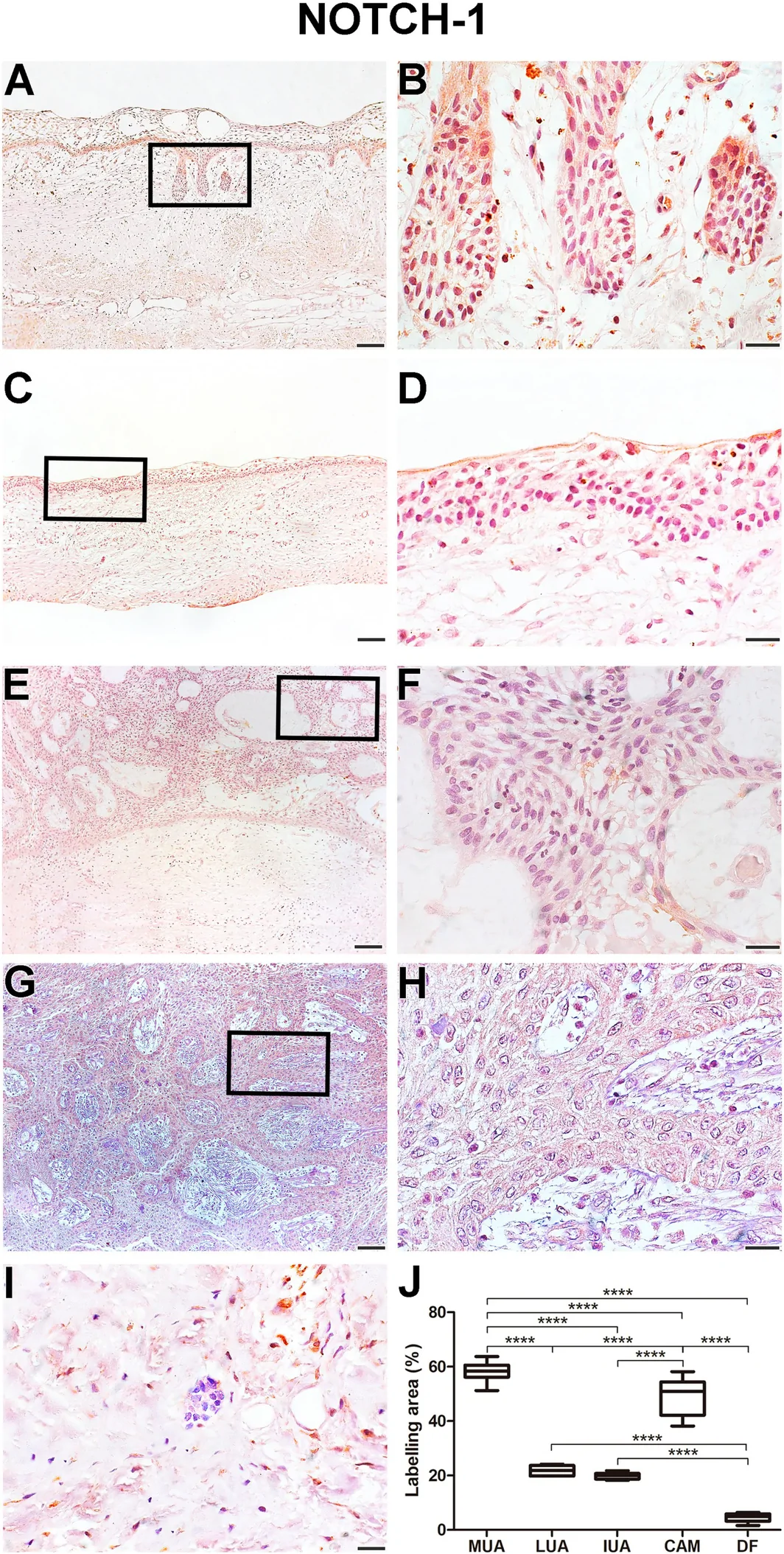
The immunoexpression of NOTCH‐1 is higher in MUA (A, B) compared to samples of LUA (C, D), IUA (E, F), CAM (G, H), and DF (I). The markings were predominantly observed in the cytoplasm of tumor parenchyma cells. Scale bars: 20 and 100 μm. Comparison of NOTCH‐1 immunoexpression between MUA, LUA, IUA, CAM, and DF samples; ****p < 0.0001, ***p < 0.001, **p < 0.01, *p < 0.05 (J).

**FIGURE 2:**
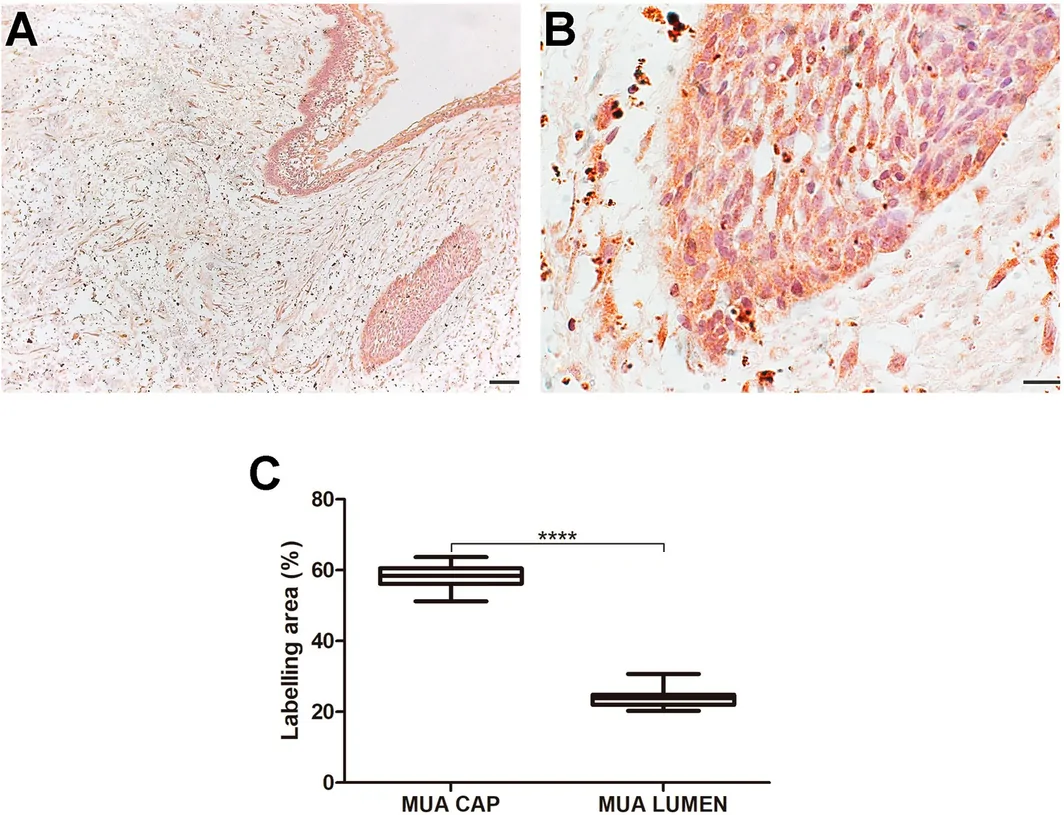
The immunostaining of NOTCH‐1 is higher in (B) neoplastic cells of MUA in the cystic capsule region compared to (A) neoplastic cells of MUA in the lumen region. Scale bars: 20 and 100 μm. (C) Comparison of NOTCH‐1 immunoexpression between the capsule and lumen areas of MUA; ****p < 0.0001.

MUA exhibited a higher ADAM‐12 immunoexpression rate compared to LUA (*p* = 0.015) and IUA (*p* < 0.001). No statistical difference was observed when comparing ADAM‐12 immunoexpression in the neoplastic cells of the cystic capsule of MUA with the neoplastic cells of CAM. Neoplastic cells in the cystic capsule of MUA showed a higher percentage of ADAM‐12 expression compared to the lumen (*p* < 0.0001). ADAM‐12 staining was observed in the cytoplasm and cell membrane of the tumor parenchyma cells. Histological images and the analysis of the results comparing ADAM‐12 protein between the UA, CAM, and DF groups are presented in Figure 3, and the comparison of the immunostaining rate of this protein between the neoplastic cell regions in the capsule and lumen of MUA can be seen in Figure 4.

**FIGURE 3:**
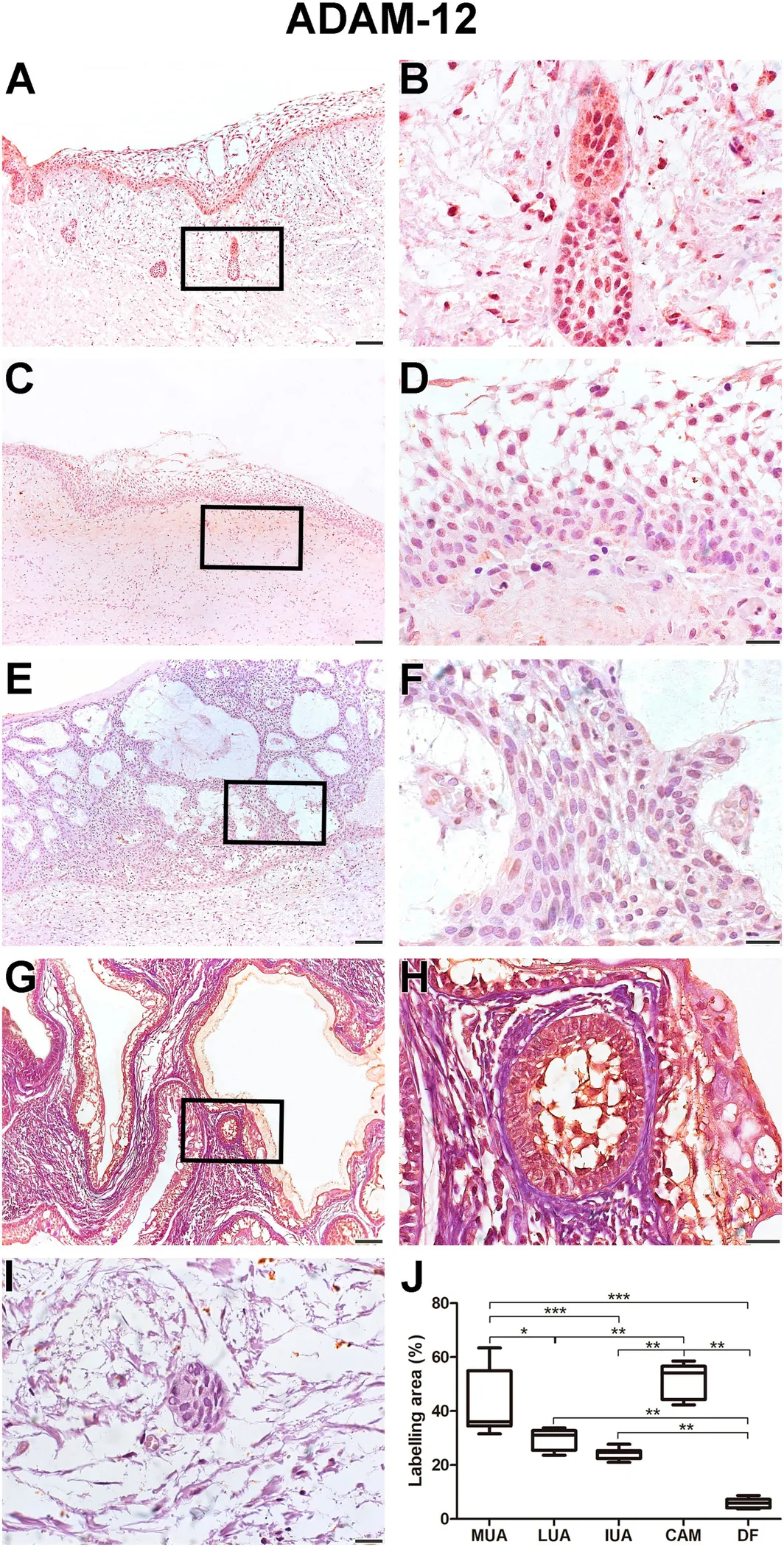
The immunoexpression of ADAM‐12 is higher in MUA (A, B) compared to samples of LUA (C, D), IUA (E, F), and DF (I). No statistical difference was observed when comparing ADAM‐12 immunoexpression in the neoplastic cells of the cystic capsule of MUA (A, B) with the cells of CAM (G, H). The staining was predominantly observed in the cytoplasm and cell membrane of the tumor parenchyma cells. Scales: 20 and 100 μm. Comparison of ADAM‐12 immunoexpression between the MUA, LUA, IUA, CAM, and DF samples; ***p < 0.001, **p < 0.01, *p < 0.05 (J).

**FIGURE 4:**
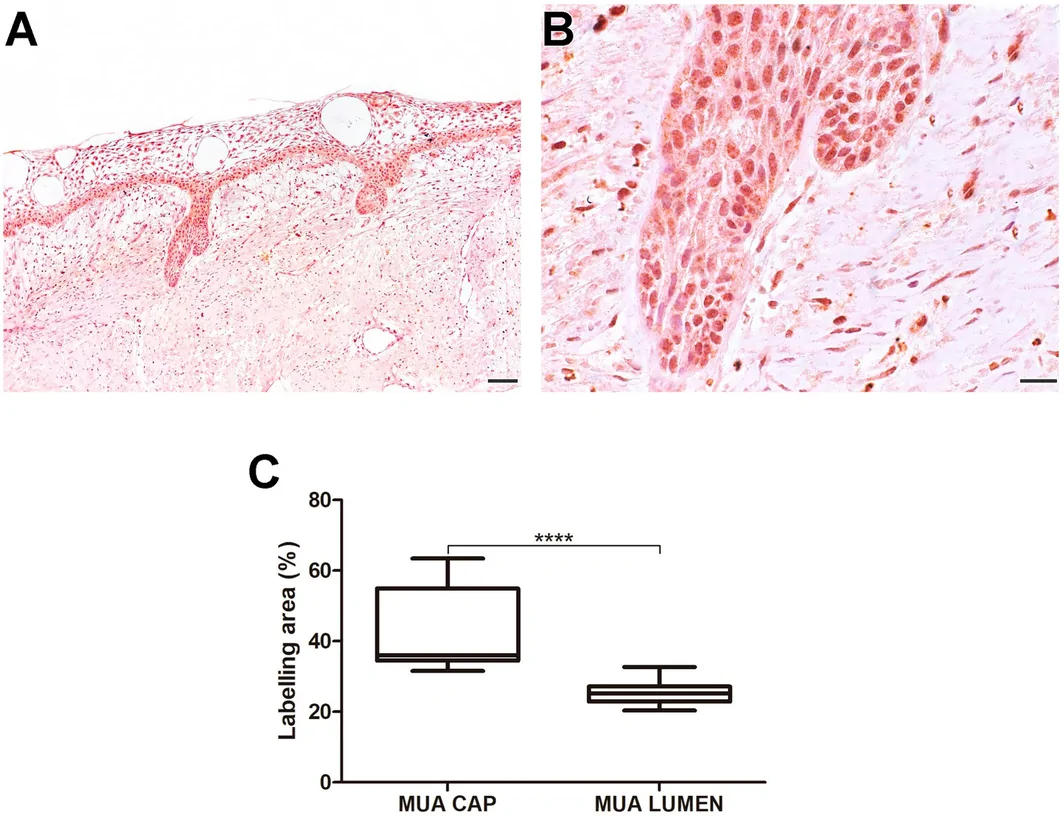
The immunoexpression of ADAM‐12 is higher in (B) neoplastic cells of MUA in the cystic capsule region compared to (A) neoplastic cells of MUA in the lumen region. Scale: 20 and 100 μm. (C) Comparison of ADAM‐12 immunoexpression between the capsule and lumen areas of MUA; ****p < 0.0001.

MUA exhibited a higher rate of HIF‐1α immunoexpression compared to LUA (*p* < 0.0001) and IUA (*p* < 0.0001). No statistical difference was observed when comparing the immunoexpression of HIF‐1α in the neoplastic cells of the MUA cystic capsule with the cells of CAM. Neoplastic cells in the cystic capsule of MUA showed a higher percentage of HIF‐1α expression compared to those in the lumen (*p* < 0.001). The HIF‐1α staining was observed both in the nucleus and the cytoplasm of tumor parenchymal cells. Figure 5 presents histological images and comparative analyses of HIF‐1α expression among the UA, CAM, and DF groups, while Figure 6 shows the immunoexpression differences between neoplastic cell regions in the capsule and lumen of MUA.

**FIGURE 5:**
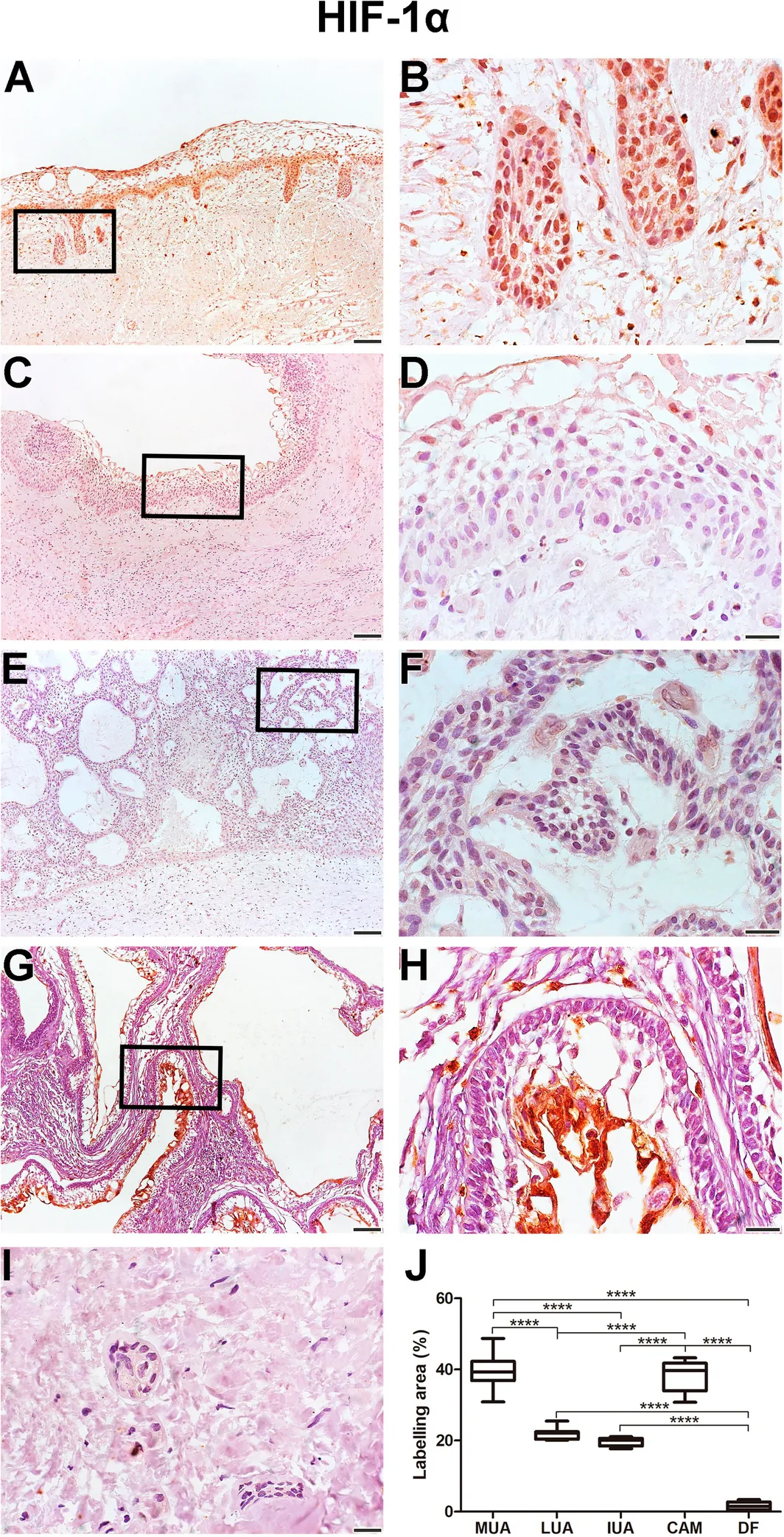
The immunoexpression of HIF‐1α is higher in MUA (A, B) compared to samples of LUA (C, D), IUA (E, F), CAM (G, H) and DF (I). No statistically significant difference was observed when comparing HIF‐1α immunoexpression in the neoplastic cells of the MUA cystic capsule with the cells of the CAM. Staining was predominantly observed in the nuclei of tumor parenchyma cells and in the cytoplasm. Scale bars: 20 μm and 100 μm. Comparison of HIF‐1α immunoexpression among MUA, LUA, IUA, CAM, and DF samples, **** *p* < 0.0001, *** *p* < 0.001, ** *p* < 0.01, * *p* < 0.05 (J).

**FIGURE 6:**
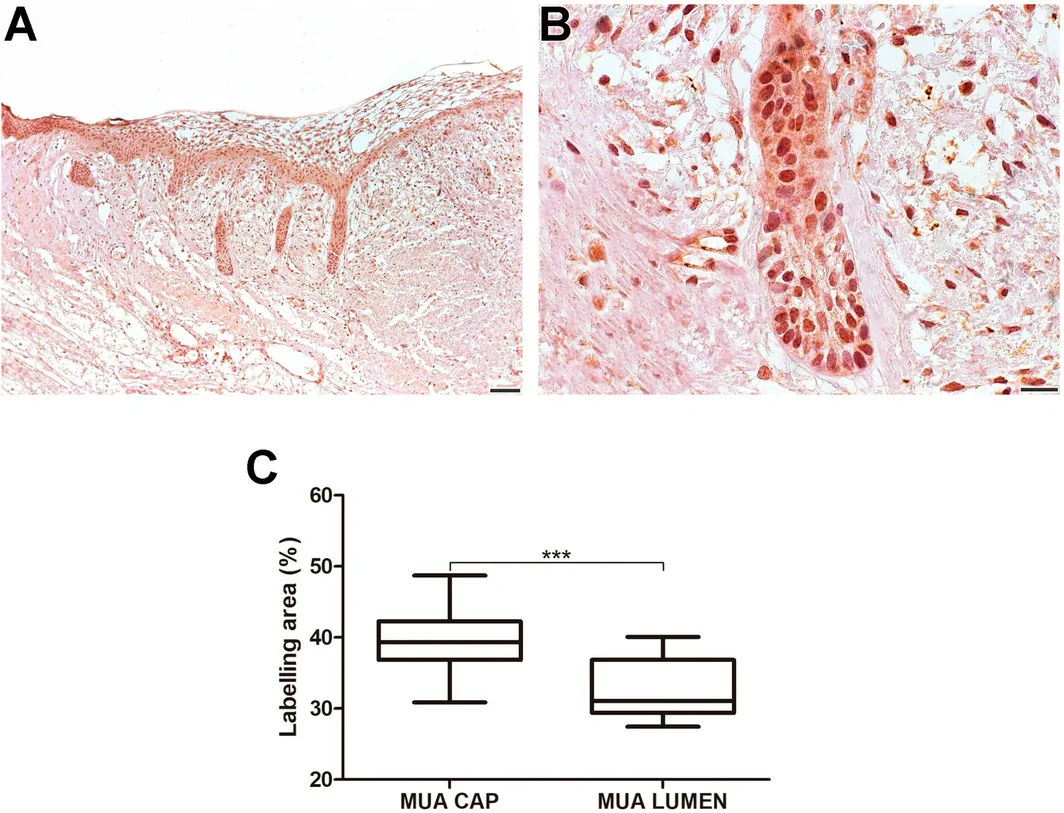
HIF‐1α immunostaining is higher in (B) neoplastic cells of MUA in the cystic capsule region compared with (A) neoplastic cells of MUA in the luminal region. Scale bars: 20 μm and 100 μm. (C) Comparison of HIF‐1α immunoexpression between the capsule and luminal areas of MUA, *** *p* < 0.001.

MUA had a higher rate of HB‐EGF immunoexpression than LUA (*p* < 0.0001) and IUA (*p* < 0.0001). No statistical difference was found when comparing the HB‐EGF immunoexpression in the neoplastic cells of the MUA cystic capsule with those of CAM.

The neoplastic cells in the cystic capsule of MUA exhibited a higher percentage of HB‐EGF expression compared to those in the lumen (*p* < 0.0001). The HB‐EGF staining was predominantly observed in the cytoplasm and cell membrane of the tumor parenchyma cells. Histological images and comparative analyses of HB‐EGF expression across the UA, CAM, and DF groups are presented in Figure 7, while Figure 8 illustrates the immunostaining differences between neoplastic cell regions in the capsule and lumen of MUA.

**FIGURE 7:**
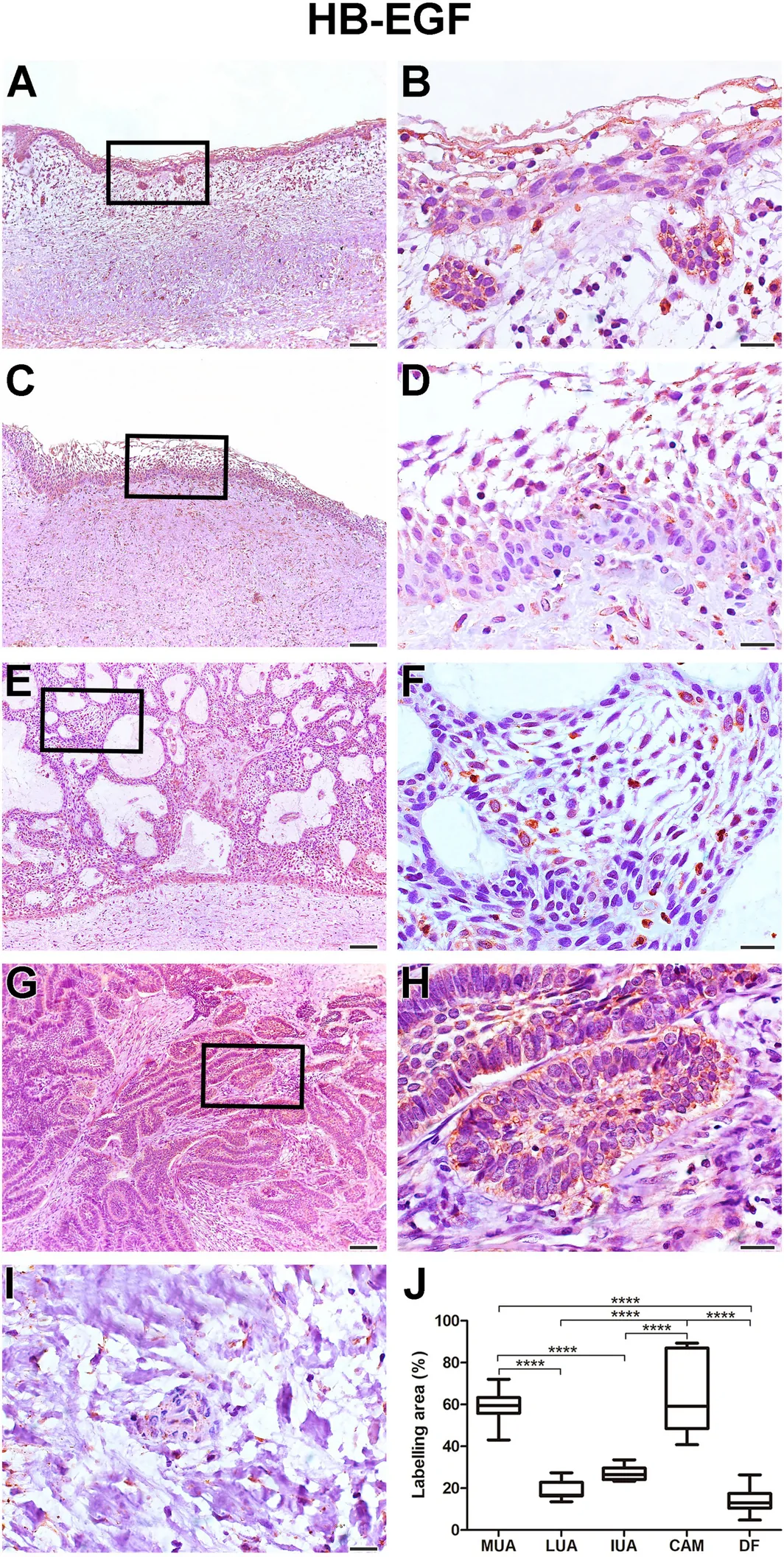
The immunoexpression of HB‐EGF is higher in MUA (A, B) compared to samples of LUA (C, D), IUA (E, F), and DF (I). No statistical difference was observed when comparing the immunoexpression of HB‐EGF in the neoplastic cells of the MUA cystic capsule (A, B) with those of CAM (G, H). Staining was predominantly observed in the cytoplasm and cell membrane of the tumor parenchyma cells. Scales: 20 and 100 μm. Comparison of HB‐EGF immunoexpression between MUA, LUA, IUA, CAM, and DF samples; ****p < 0.0001, ***p < 0.001, **p < 0.01, *p < 0.05 (J).

**FIGURE 8:**
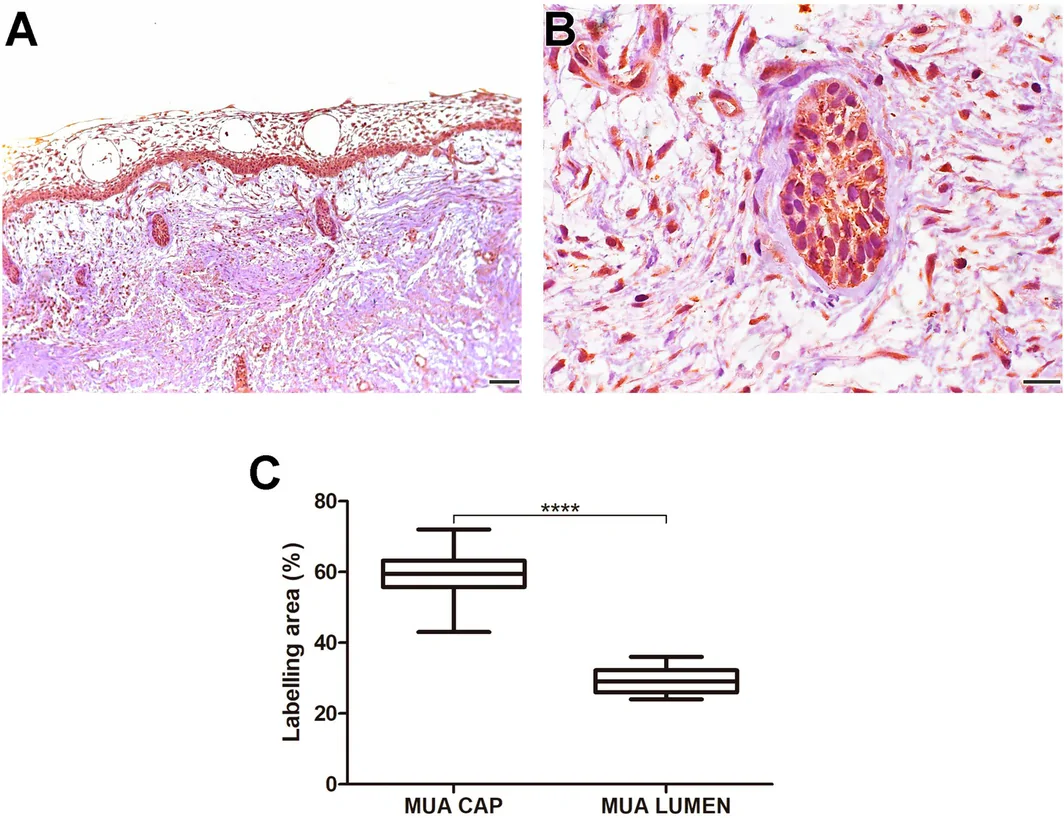
The immunoexpression of HB‐EGF is higher in (B) neoplastic cells of MUA in the cystic capsule region compared to (A) neoplastic cells of MUA in the lumen region. Scales: 20 and 100 μm. (C) Comparison of HB‐EGF immunoexpression between the capsule and lumen areas of MUA; ****p < 0.0001.

Measures of central tendency, standard deviation, and *p* values are presented in Tables 2 and 3.

**TABLE 2:** Comparison of immunoexpression of NOTCH‐1, ADAM‐12, HIF‐1α, and HB‐EGF in the epithelial cells between groups of mural unicystic ameloblastoma, luminal unicystic ameloblastoma, intraluminal unicystic ameloblastoma, conventional ameloblastoma, and dental follicle.

Proteins	MUA (*n* = 14)		LUA (*n* = 7)		IUA (*n* = 8)		CAM (*n* = 9)		DF (*n* = 7)		*p*									
	Mean	SD	Mean	SD	Mean	SD	Mean	SD	Mean	SD	MUA × LUA	MUA × IUA	MUA × CAM	MUA × DF	LUA × IUA	LUA × CAM	LUA × DF	IUA × CAM	IUA × DF	CAM × DF
NOTCH‐1	58.21	±3.522	21.84	±1.692	19.92	±1.177	48.23	±7.099	4.62	±1.609	< 0.0001	< 0.0001	< 0.0001	< 0.0001	0.865	< 0.0001	< 0.0001	< 0.0001	< 0.0001	< 0.0001
ADAM‐12	43.00	±11.2	29.70	±3.841	24.34	±2.089	51.71	±6.335	5.862	±1.773	0.015	< 0.001	0.395	< 0.001	0.122	0.008	0.008	0.005	0.005	0.003
HIF‐1α	39.56	±4.958	22.07	±1.743	19.73	±1.210	38.23	±4.521	1.672	±1.169	< 0.0001	< 0.0001	0.903	< 0.0001	0.705	< 0.0001	< 0.0001	< 0.0001	< 0.0001	< 0.0001
HB‐EGF	44.57	±5.019	18.93	±4.696	27.08	±3.608	63.37	±18.51	14.03	±5.929	< 0.0001	0.005	< 0.0001	< 0.01	0.559	< 0.0001	0.841	< 0.0001	0.086	< 0.0001

*Note:* ANOVA, followed by Bonferroni multiple comparison test. Kruskal–Wallis test, followed by Dunn's multiple comparison test (*p* < 0.05).

Abbreviations: CAM, conventional ameloblastoma; DF, dental follicle; IUA, intraluminal unicystic ameloblastoma; LUA, luminal unicystic ameloblastoma; MUA, mural unicystic ameloblastoma; SD, standard deviation.

**TABLE 3:** Comparison of immunoexpression of NOTCH‐1, ADAM‐12, HIF‐1α, and HB‐EGF between the epithelial cells of mural unicystic ameloblastomas between capsule, lumen, and conventional ameloblastoma.

Proteins	MUA capsule		MUA lumen		CAM		MUA capsule × MUA lumen	MUA capsule × CAM	MUA lumen × CAM
	Mean	SD	Mean	SD	Mean	SD	*p*	*p*	*p*
NOTCH‐1	60.66	±5.896	34.55	±5.975	48.23	±7.099	< 0.0001	< 0.0001	< 0.0001
ADAM‐12	43.00	±11.20	25.32	±3.112	51.71	±6.335	0.0002	0.7678	< 0.0001
HIF‐1α	39.56	±4.958	32.67	±4.190	38.23	±4.521	0.0031	1.0000	0.0284
HB‐EGF	59.36	±7.132	29.50	±3.878	63.37	±18.51	< 0.0001	1.0000	< 0.0001

*Note:* ANOVA, followed by Bonferroni multiple comparison test. Kruskal–Wallis test, followed by Dunn's multiple comparison test (*p* < 0.05).

Abbreviations: CAM, conventional ameloblastoma; MUA, mural unicystic ameloblastoma; SD, standard deviation.

## 4 Discussion

Using immunohistochemistry, it was observed that samples from MUA, LUA, IUA, CAM, and DF expressed the proteins NOTCH1, HIF‐1α, ADAM‐12, and HB‐EGF. Furthermore, elevated immunoexpression was noted in MUA, LUA, IUA, and CAM when compared to the control group, DF. This was expected, as these proteins are associated with neoplastic invasion, which is not the case for the dental follicle.

Both MUA and CAM exhibited high expression of the proteins HIF‐1α, ADAM‐12, and HB‐EGF. However, only NOTCH‐1 showed greater immunoexpression in the tumor cells of the cystic capsule of MUA when compared to those of CAM.

These results possibly indicate the involvement of these proteins in the local invasiveness of MUA. Our primary focus was to associate the studied proteins with hypoxia, as the literature provides strong evidence linking oxygen deficient microenvironments to the pathogenesis of certain benign and malignant tumors (Díaz et al. 2013; Miranda da Costa et al. 2019; de Mendonça et al. 2020; Adelman et al. 2000). Previous studies by our group also demonstrated the immunoexpression of tumor proliferation and invasion‐related proteins in unicystic and conventional ameloblastomas, reinforcing the notion that mural subtypes display a more aggressive biological behavior (da Silveira et al. 2025; Vieira Costa et al. 2024).

In our findings, we observed overexpression of NOTCH‐1, with predominant cytoplasmic staining and mild nuclear staining in the tumor epithelium of MUA and CAM. NOTCH‐1 is known to be involved in the invasive process of certain benign and malignant neoplasms (Da Costa et al. 2016; Zhu et al. 2013; Alniaimi et al. 2015; An et al. 2015). Additionally, it has been implicated in pathways related to invadopodia formation and cellular invasion (Díaz et al. 2013), although immunohistochemical expression alone does not demonstrate the assembly of functional invasive structures.

One of the key factors responsible for stabilizing the intracellular response triggered by NOTCH‐1 is HIF‐1α (Gustafsson et al. 2005; Chen et al. 2010; Pietras et al. 2011). HIF‐1α is classically recognized as a transcription factor induced by hypoxia (Wang and Semenza 1995; Greer et al. 2012). When active, it is localized in the nucleus (Swartz et al. 2015), facilitating the transcription of hypoxia‐responsive genes (Chen et al. 2010; Pietras et al. 2011; Wang and Semenza 1995). In our analyses, we observed high HIF‐1α immunoexpression in MUA, with intense nuclear staining in tumor epithelial cells located in the cystic capsule, as well as nuclear and cytoplasmic staining in cells adjacent to or in close contact with cystic structures. These findings were similar in CAM.

In tumorigenesis, hypoxia creates a critical condition for the progression and adaptation of neoplastic cells (Adelman et al. 2000). The presence of hypoxia has been reported using endogenous hypoxia biomarkers (Nguyen et al. 2013). Among these, HIF‐1α has been described as the primary biomarker, as it is not only induced by hypoxia but also triggers the transcription of genes that lead to the synthesis of other biomarkers (Li et al. 2011). The presence of HIF‐1α in MUA is essential for suggesting intratumoral hypoxia in specific areas, such as the capsule, where staining is predominantly nuclear, and in the epithelium adjacent to cystic formations, where both nuclear and cytoplasmic staining are observed.

In MUA samples with high levels of HIF‐1α, NOTCH‐1 signaling is likely stabilized, triggering the transcription of various genes, including ADAM‐12 (Shao et al. 2014; Abram et al. 2003). Our results demonstrated elevated ADAM‐12 expression in MUA samples, with intense cytoplasmic and membrane immunostaining, particularly in neoplastic epithelial cells of the cystic capsule.

Another key point to clarify is that ADAM‐12 can initiate signaling through its cytoplasmic domain, allowing interactions with substrates from the SRC family, particularly Tks‐5 (Zhao and Qin 2019). Previous analyses have demonstrated the expression of proteins associated with invadopodia formation, including Tks‐4, Tks‐5, cortactin, and MT1‐MMP in MUA. Therefore, higher ADAM‐12 expression may be associated with increased activation of invasion‐related signaling pathways, suggesting an immunophenotypic profile compatible with enhanced invasive potential of this neoplasm rather than direct evidence of invadopodia formation.

Studies have investigated hypoxia and its relationship to the local invasiveness of odontogenic cysts and tumors. One study concluded that HIF‐1α, a well‐established hypoxia marker, is likely associated with the invasive mechanism of conventional ameloblastoma. The results demonstrated increased expression of this protein in CAM, particularly in the tumor center, indicating the presence of intratumoral hypoxia and in the epithelium adjacent to cystic formations, suggesting a link between hypoxia, apoptosis of neoplastic cells, and subsequent cystic formation.

Additionally, it has been proposed that increased expression of NOTCH‐1, HIF‐1α, ADAM‐12, and HB‐EGF may be associated with molecular pathways related to cellular invasion. In our study, HIF‐1α expression was observed in the analyzed samples, particularly in neoplastic epithelial cells lining the cystic cavity and within the capsule. The higher expression in cystic capsule cells may suggest localized hypoxic conditions, which, together with the increased expression of NOTCH‐1, ADAM‐12, and HB‐EGF in MUA, indicates an immunophenotypic profile compatible with increased invasive potential (Da Costa et al. 2016; Miranda da Costa et al. 2019; de Mendonça et al. 2020).

ADAM‐12 plays a crucial role in cleaving specific growth factors from their ectodomains, including HB‐EGF, thereby stimulating key cellular invasion processes such as proliferation, differentiation, and migration (Abram et al. 2003).

Another important consideration is the proximity of neoplastic cells in the cystic capsule to the resorbing bone surrounding the lesion. Bone resorption can release growth factors and cytokines stored in the bone matrix, which may influence the expression of the studied molecules. However, since the present study did not include specific analyses of the bone microenvironment, this interpretation should be regarded as a theoretical hypothesis based on previously reported mechanisms rather than direct evidence. Therefore, this possibility warrants further investigation in studies specifically designed to evaluate tumor–bone interactions (Zhu et al. 2022; Lin and Wu 2020).

Additionally, the stromal immunoreactivity observed, particularly for ADAM‐12 and HB‐EGF, may be relevant to the invasive behavior of the lesion. The tumor microenvironment plays a critical role in neoplastic progression, as stromal fibroblasts can secrete growth factors, cytokines, and proteolytic enzymes that facilitate extracellular matrix remodeling and tumor cell migration (Witz 2009; Timperi et al. 2024). ADAM‐12 is known to participate in matrix degradation and growth factor activation, while HB‐EGF can mediate epithelial–stromal signaling pathways associated with tumor progression (Abram et al. 2003). Therefore, the stromal staining observed in this study is unlikely to represent nonspecific background and may instead reflect a reactive microenvironment that supports invasion‐related processes. Nevertheless, since the present analysis was based on immunohistochemical expression, further functional studies are necessary to clarify the precise contribution of stromal cells.

In summary, our findings suggest that NOTCH‐1, HIF‐1α, ADAM‐12, and HB‐EGF are expressed in MUA in a pattern compatible with increased invasive potential. The elevated expression of these proteins, particularly in the cystic capsule, may indicate the presence of hypoxia‐related signaling pathways. Although these molecules have been implicated in invasion‐associated mechanisms in the literature, the present study demonstrates immunohistochemical expression only and does not establish direct functional interactions or confirm invadopodia formation. Nevertheless, the observed immunophenotypic profile supports the hypothesis that these pathways may contribute to the more aggressive biological behavior reported for mural subtypes.

An important limitation of this study is the small sample size, which may compromise the robustness of the results and their generalizability to a larger population of unicystic ameloblastoma cases. Furthermore, although immunohistochemical analysis is essential for evaluating the expression of the studied proteins, it does not allow for determining direct functional relationships between them. Another limitation of the study was the difficulty in obtaining complete clinical and radiographic information, which restricted the possibility of correlating protein expression with variables related to the biological behavior of the lesions. Additional studies with functional approaches, such as in vitro assays and experimental models, would be necessary to confirm the role of these proteins in the invasiveness of MUA.

## 5 Conclusion

The neoplastic cells in the cystic capsule of MUA exhibited higher immunoexpression levels of proteins associated with tumor hypoxia (HIF‐1α, NOTCH‐1, ADAM‐12, and HB‐EGF) compared to neoplastic cells located in the lumen of MUA and cells from LUA and IUA. Both MUA and CAM showed high expression of HIF‐1α, ADAM‐12, and HB‐EGF. However, only NOTCH‐1 demonstrated significantly higher immunoexpression in the tumor cells of the cystic capsule of MUA compared to CAM.

These findings suggest that tumor cells in closer proximity to the bone resorption area may be influenced by molecules released during bone resorption. This process could lead to the increased expression of hypoxia‐related proteins and promote invadopodia formation, resulting in more aggressive biological behavior in MUA compared to other UA subtypes.

## Author Contributions

**Gabriela Cristina Avertano Rocha da Silveira:** formal analysis, methodology, writing – original draft. **Rebeca Vieira Costa:** formal analysis, writing – original draft. **Flavia Letícia Magalhães Lemos:** formal analysis, writing – original draft. **Antonia Taiane Lopes de Moraes:** formal analysis, writing – review and editing. **Maria Sueli da Silva Kataoka:** data curation, writing – review and editing. **Vanessa Morais Freitas:** methodology, writing – review and editing. **Douglas Magno Guimarães:** data curation, writing – review and editing. **Ana Paula Neutzling Gomes:** data curation, writing – review and editing. **Adriana Etges:** data curation, writing – review and editing. **Fabricio Passador Santos:** data curation, writing – review and editing. **Vera Cavalcanti de Araújo:** methodology, writing – review and editing. **Andrew Mark Smith:** methodology, writing – review and editing. **Sérgio de Melo Alves Júnior:** data curation, writing – review and editing. **Ruy Gastaldoni Jaeger:** conceptualization, writing – review and editing. **João de Jesus Viana Pinheiro:** conceptualization, data curation, methodology, writing – review and editing.

## Funding

This study was privately funded by the authors.

## Ethics Statement

This study was conducted in accordance with the Declaration of Helsinki and approved by the Research Ethics Committee on Human Subjects of the Institute of Health Sciences at the Federal University of Pará (protocol number 4.570.860).

## Consent

Informed consent was obtained from all subjects involved in the study.

## Conflicts of Interest

The authors declare no conflicts of interest.

## Data Availability

The data that support the findings of this study are available on request from the corresponding author. The data are not publicly available due to privacy or ethical restrictions.
